# Patient activation and its association with symptom burden and quality of life across the spectrum of chronic kidney disease stages in England

**DOI:** 10.1186/s12882-022-02679-w

**Published:** 2022-01-26

**Authors:** Winnie Magadi, Courtney J. Lightfoot, Katherine E. Memory, Shalini Santhakumaran, Sabine N. van der Veer, Nicola Thomas, Rachel Gair, Alice C. Smith

**Affiliations:** 1 UK Renal Registry, Brandon House Building 20A1, Southmead Road, Bristol, BS34 7RR UK; 2 Leicester Kidney Lifestyle Team, Department of Health Sciences, University of Leicester, Leicester, UK; 3 Centre for Health Informatics, Division of Informatics, Imaging and Data Sciences, Manchester Academic Health Science Centre, The University of Manchester, Manchester, UK; 4 School of Health and Social Care, London South Bank University, London, UK

**Keywords:** Patient activation, Symptom burden, Quality of life, Chronic kidney disease, Patient reported outcomes

## Abstract

**Background:**

The knowledge, skills, and confidence to manage one’s own health is termed patient activation and can be assessed using the Patient Activation Measure (PAM). This measure is increasingly recommended for use in chronic kidney disease (CKD), but there is a need to better understand patient activation within this population. This work aimed to explore the association of PAM with patient-reported outcomes, namely symptom burden and health-related quality of life (HRQoL), to understand the relationship between patient activation and outcomes which are of importance to people with CKD.

**Methods:**

Non-dialysis, dialysis, and kidney transplant patients from 14 renal units across England completed a survey comprising questionnaires assessing patient activation, symptom burden, and HRQoL.

Latent class analysis (LCA) was used to determine HRQoL and symptom burden subgroups in the data. Multinomial logistic regression analyses were performed to investigate the associations between patient activation and symptom burden and HRQoL classes separately, adjusting for age, gender, ethnicity, deprivation and treatment modality.

**Results:**

Three thousand thirteen participants (mean age 61.5 years, 61.8% males, and 47% haemodialysis) were included in the analysis. Patient activation was strongly associated with both the HRQoL and symptom burden classes identified, with highly activated patients more likely to report higher HRQoL (*P* = < 0.0001; OR 29.2, 95% CI 19.5–43.9) and fewer symptoms (*P* = < 0.0001; OR 25.9, 95% CI 16.8–40.2).

**Conclusion:**

Lower activation levels are associated with a higher symptom burden and reduced HRQoL across the trajectory of CKD stages and treatment modalities. Therefore, targeted and holistic self-management support focussing on improving activation may have the potential to improve aspects of health experience which are valued by individuals living with kidney disease.

**Supplementary Information:**

The online version contains supplementary material available at 10.1186/s12882-022-02679-w.

## Introduction

The ability to manage one’s own health is a key determinant in improving long-term health outcomes and quality of life (QoL) for a variety of chronic health conditions [1]. The concept of patient activation describes the knowledge, skills and confidence to manage one’s own health and healthcare [2, 3]. The most widely used tool for assessment of activation is the Patient Activation Measure (PAM) [2]. The PAM categorises individuals into one of four activation levels ranging from Level 1 (passive and lacking knowledge and skills) to Level 4 (active, well-informed and competent). Higher levels of activation are often associated with lower healthcare costs [4] and improved health outcomes [5, 6]. Individuals described as being highly activated are also more likely to participate in healthy lifestyle behaviours [7] and access health services including check-ups, screening and immunisations [3, 8].

Patient activation is increasingly acknowledged to underpin self-management [9]. In order to effectively manage long-term conditions such as chronic kidney disease (CKD), individuals are required to take an active role in their health using skills developed through information and support obtained from various educational and healthcare resources [10]. In CKD, poor engagement with self-management behaviours are associated with poor clinical outcomes such as progression to end-stage kidney disease (ESKD), cardiovascular disease, and death [11]. Patient activation has been used to tailor self-management support interventions to improve behavioural and health-related outcomes for patients with CKD [12]. The aim of increasing activation levels has also been incorporated into policies involving CKD populations [13].

With the application of the PAM in CKD being increasingly recommended [9], and recent validation in CKD [14], there is a need to better understand the factors which influence or are influenced by patient activation within this population. While clinical and cost outcomes are important to clinicians, healthcare provider organisations and policy makers, they are not necessarily rated as the most valued considerations for those living with the disease. There is now increasing global recognition of the need to incorporate the patient perspective in research and care planning and delivery in order to achieve improvements that provide genuine benefit in aspects of health and life which matter to the individual [15]. Patient-reported outcomes (PROs) such as health-related QoL (HRQoL) and symptom burden are such priorities identified by kidney patients themselves [16]. The need for a more personalised and patient-centred approach to health and care has been recognised in the NHS long-term plan with the introduction of a Comprehensive Model for Personalised Care [17]; this model of care is intended to support self-management, improve health and wellbeing outcomes, and quality of care, particularly for those with long-term conditions such as CKD [17]. Given the increasing prioritisation of patient activation and PROs, such as HRQoL and symptom burden, there is a need to understand the relationship between these factors to improve patient care and develop future health-promotion and self-management interventions.

The Transforming Participation in CKD (TP-CKD) programme gathered Patient Reported Outcome Measure (PROM) data from an English population, comprising people with CKD, including those not requiring dialysis, receiving dialysis and kidney transplant recipients [18]. The current study aimed to explore activation levels and associated factors including PROs in the TP-CKD cohort. It was hypothesised that more activated individuals living with CKD would have a higher HRQoL and lower symptom burden than less activated individuals.

## Methods

### Study design and setting

This study utilised secondary data from the UK Renal Registry (UKRR) (ref: UKRR ILD24) collected as part of the *national TP-CKD service evaluation programme.* The programme, involving patients, carers, clinicians and commissioners, was based on a multi-centre, longitudinal cohort of CKD patients, either on renal replacement therapy (RRT; dialysis or transplantation) or not on RRT (termed non-dialysis), managed in secondary care. Participants were recruited from 14 renal units across England and completed a paper survey distributed by local renal teams between December 2015 and December 2017 [18]. Each survey comprised three questionnaires assessing patient activation, symptom burden and HRQoL. Once completed, the surveys were scanned by the UKRR into electronic format to allow linkage to other UKRR data items used in the study.

### Participants

Inclusion criteria included: (1) patients with any stage of CKD or receiving any form of RRT in renal clinics in secondary care; (2) ≥18 years; and 3) implicitly consented to their patient-reported outcome data being held by the UKRR.

### Demographics

Demographics (date of birth, gender, ethnicity and index of multiple deprivation area), as well as treatment modality, were obtained by linking the participant’s survey data to UKRR data using their unique NHS number. The UKRR only has full coverage of people on RRT, thus, individuals lacking information about RRT modality were assigned to the “non-dialysis” cohort, although no data on their stage of CKD was available.

### Patient activation measure (PAM)

The 13-item PAM version, which draws on concepts such as health locus of control and self-efficacy in managing health behaviours, was used to assess patient activation [19]. Responses for each item were given on a 4-point Likert scale ((1 strongly disagree to 4 strongly agree, with an additional “not applicable” option), assigning scores from 1 to 4 respectively. Raw scores were then converted to a scale of 0–100 and an activation level of 1 to 4 indicating low to high activation. The activation groups are described as follows: Level 1 (score ≤ 47) consists of patients who do not believe they have an important role in their health; Level 2 (score 47.1–55.1) describes patients lacking in confidence or knowledge to take action; Level 3 (score 55.2–67.0) comprises patients starting to take action while Level 4 (score ≥ 67.1) includes patients who maintain active behaviour. The PAM has recently been validated in the CKD population [14].

### EuroQol- 5 dimension (EQ-5D-5L)

HRQoL was assessed using the EQ-5D-5L questionnaire, which measures 5 dimensions: mobility, self-care, usual activities, pain/discomfort, and anxiety/depression [20]. This is a widely used, validated measure of health status that can be standardised to different populations [21]. Participants rated each dimension on a scale from 1 (no problem) to 5 (unable). Responses to the five dimensions were combined into a 5-digit score to describe an individual’s health state, with ‘11111’ indicating ‘no problems at all’ and ‘55555’ indicating ‘extreme problems’ [20]. A single utility score was then assigned to each combination using a scoring algorithm and based on the UK value set.

### Palliative care outcome scale-symptom (POS-S) renal

Symptom burden was assessed using the 17-symptom POS-S Renal questionnaire [22]. Respondents indicated the extent to which they were bothered by each symptom over the last week on a scale from 0 (not at all) to 4 (overwhelmingly).

### Statistical analysis

Descriptive analyses were performed for demographic parameters to compare characteristics of individuals in each of the four patient activation groups. Inferential statistics were also performed for outcome variables, including calculating mean scores for all dimensions of EQ-5D-5L and all POS-S Renal items and comparing them among the groups.

Latent class analysis (LCA) was utilised to determine whether HRQoL and symptom burden subgroups existed in the data, and if so, identify classes that best described the data. Latent class analysis is a statistical modelling approach which aims to find heterogeneity within the population by classifying individuals into unobserved groupings (latent classes) based on similar patterns of observed cross-sectional and/or longitudinal data [23]. As such, the goal is to probabilistically assign individuals into subpopulations by inferring each individual’s membership to latent classes from the data. Thus, we conceptualized HRQoL and symptom burden as forming distinct categories or typologies as the use of raw scores may mask important differences among patients i.e. classes who self-report different types of limitations. We ran a 2, 3 and 4-class model to determine how many classes best described the subgroups in the population. The Bayesian Information Criteria (BIC) was used to determine goodness of fit [24]. These analyses were conducted using a SAS procedure developed by The Methodology Centre, PROC LCA [25].

Multinomial regression models were then developed to investigate: 1) the associations between patient activation and HRQoL classes and 2) the associations between patient activation and symptom burden classes. To handle missing data, we used multiple imputation with fully conditional specification, assuming the data were missing at random. We carried out 20 imputations using all available data on activation levels, symptom scores, HRQoL scores, and clinical and demographic data as predictor variables. Participants who had missing data on all the HRQoL and/or all the symptom burden items were excluded from the imputation and analyses. We checked that the distributions and correlations between variables were consistent between imputed and observed data. For both models, we controlled for the following factors; age, gender, ethnicity, treatment modality and index of multiple deprivation area quintile [26] (proxy of socio-economic status derived from postcode, with higher quintiles representing more social deprivation). The PROC MIANALYZE procedure in SAS was then utilised to obtain the pooled parameter estimates and the variance information from the 20 imputations we ran prior [27]. We used SAS version 9.4 for all analyses.

## Results

### Patient characteristics

A total of 312 participants were excluded due to refused consent (*n* = 1), aged < 18 (*n* = 8), missing all symptom burden questions (*n* = 243) and missing all EQ-5D-5L questions (*n* = 60). Thus, the final study sample comprised 3013 participants who had similar demographic characteristics to the overall CKD and RRT population in the UK in 2016 (see Additional File 1 Table s2). Participant characteristics are displayed in Table 1. In summary, the mean age was 61.5 years, 61.8% were male, 81% white and 47% on haemodialysis. A third (34%) of participants were categorised into PAM Level 3 (i.e. taking action to manage their own health). Mean scores for HRQoL and symptom burden dimensions were generally highest in the least activated group (PAM Level 1) and lowest in the most activated group (PAM Level 4) *as shown in* Table 2*.*

**Table 1:** Baseline characteristics, presented with column percentages unless indicated otherwise

^a^Total N	All	PAM Level 1	PAM Level 2	PAM Level 3	PAM Level 4	Missing
	3013 (100)	756 (25.1)	571 (19.0)	1023 (34.0)	526 (17.5)	137 (4.5)
**Covariates**						
Treatment type						
Haemodialysis	1415 (47.0)	508 (67.2)	284 (49.7)	417 (40.8)	149 (28.3)	57 (41.6)
Peritoneal dialysis	122 (4.0)	22 (2.9)	25 (4.4)	46 (4.5)	27 (5.1)	2 (1.5)
Transplant	816 (27.1)	90 (11.9)	120 (21.0)	321 (31.4)	248 (47.1)	37 (27.0)
Non-dialysis	660 (21.9)	136 (18.0)	142 (24.9)	239 (23.4)	102 (19.4)	41 (29.9)
Age (years)						
18–44	496 (16.5)	93 (12.3)	78 (13.7)	176 (17.2)	132 (25.1)	17 (12.4)
45–54	524 (17.4)	100 (13.2)	86 (15.1)	198 (19.4)	124 (23.6)	16 (11.7)
55–64	650 (21.6)	165 (21.8)	125 (21.9)	212 (20.7)	115 (21.9)	33 (24.1)
65–74	663 (22.0)	182 (24.1)	139 (24.3)	225 (22.0)	88 (16.7)	29 (21.2)
75+	678 (22.5)	216 (28.6)	143 (25.0)	211 (20.6)	67 (12.7)	41 (29.9)
Missing	2 (0.1)	0 (0)	0 (0)	1 (0.1)	0 (0)	1 (0.7)
Gender						
Male	1568 (52.0)	411 (54.4)	292 (51.1)	531 (51.9)	270 (51.3)	64 (46.7)
Female	970 (32.2)	237 (31.3)	186 (32.6)	327 (32.0)	179 (34.0)	41 (29.9)
Missing	475 (15.8)	108 (14.3)	93 (16.3)	165 (16.1)	77 (14.6)	32 (23.4)
Ethnicity						
White	2036 (67.6)	468 (61.9)	381 (66.7)	718 (70.2)	395 (75.1)	74 (54.0)
Asian	285 (9.5)	119 (15.7)	44 (7.7)	80 (7.8)	26 (4.9)	16 (11.7)
Black	148 (4.9)	42 (5.6)	38 (6.7)	41 (4.0)	18 (3.4)	9 (6.6)
Chinese	14 (0.5)	3 (0.4)	2 (0.4)	6 (0.6)	3 (0.6)	0 (0)
Other	33 (1.1)	12 (1.6)	8 (1.4)	5 (0.5)	3 (0.6)	5 (3.6)
Missing	497 (16.5)	112 (14.8)	98 (17.2)	173 (16.9)	81 (15.4)	33 (24.1)
IMD						
Quintile 1 (least deprived)	462 (15.3)	85 (11.2)	86 (15.1)	172 (16.8)	108 (20.5)	11 (8.0)
Quintile 2	506 (16.8)	104 (13.8)	93 (16.3)	177 (17.3)	113 (21.5)	19 (13.9)
Quintile 3	495 (16.4)	117 (15.5)	100 (17.5)	163 (15.9)	95 (18.1)	20 (14.6)
Quintile 4	625 (20.7)	170 (22.5)	103 (18.0)	221 (21.6)	103 (19.6)	28 (20.4)
Quintile 5 (most deprived)	886 (29.4)	272 (36.0)	184 (32.2)	277 (27.1)	98 (18.6)	55 (40.1)
Missing	39 (1.3)	8 (1.1)	5 (0.9)	13 (1.3)	9 (1.7)	4 (2.9)

^a^The percentages reported for the total number of participants in each PAM level are the row percentages

*Note*: *IMD* index of multiple deprivation

**Table 2:** Mean scores for health-related quality of life (EQ-5D-5L) dimensions and symptom burden (POS-S Renal) by patient activation level

	All	PAM Level 1	PAM Level 2	PAM Level 3	PAM Level 4	Missing
**Total N**	3013	756	571	1023	526	137
**Outcomes**						
EQ-5D-5L	Mean (SD) score ^a)^					
**All dimensions**	**2.1 (0.9)**	**2.8 (0.9)**	**2.2 (0.8)**	**1.9 (0.8)**	**1.5 (0.7)**	**2.5 (1.1)**
Mobility	2.4 (1.2)	3.2 (1.1)	2.6 (1.1)	2.2 (1.1)	1.7 (1)	2.8 (1.4)
Self-care	1.7 (1.0)	2.4 (1.2)	1.6 (0.9)	1.4 (0.8)	1.2 (0.6)	2 (1.3)
Usual activities	2.4 (1.3)	3.2 (1.2)	2.5 (1.2)	2.1 (1.2)	1.6 (1.0)	2.8 (1.5)
Pain/Discomfort	2.2 (1.1)	2.8 (1.2)	2.3 (1.1)	1.9 (1.0)	1.7 (1.0)	2.4 (1.3)
Anxiety/Depression	1.9 (1.1)	2.5 (1.2)	1.9 (1.0)	1.7 (0.9)	1.5 (0.8)	2.2 (1.4)
POS-S Renal	Mean (SD) score					
**All symptoms**	**1.7 (0.5)**	**2.0 (0.5)**	**1.7 (0.5)**	**1.6 (0.5)**	**1.5 (0.5)**	**1.9 (0.6)**
Pain	1.1 (1.1)	1.6 (1.2)	1.1 (1.1)	1.0 (1.1)	0.7 (1.0)	1.2 (1.2)
Shortness of breath	1.1 (1.1)	1.5 (1.1)	1.1 (1.0)	1.0 (1.0)	0.7 (0.9)	1.2 (1.2)
Weakness	1.7 (1.1)	2.2 (1.1)	1.7 (1.1)	1.5 (1.1)	1.1 (1.0)	1.8 (1.3)
Nausea	0.6 (0.9)	1.0 (1.1)	0.6 (0.9)	0.5 (0.8)	0.4 (0.7)	0.8 (1.0)
Vomiting	0.3 (0.7)	0.5 (0.9)	0.3 (0.7)	0.2 (0.6)	0.2 (0.6)	0.5 (0.8)
Poor appetite	0.8 (1.1)	1.2 (1.2)	0.8 (1.0)	0.7 (1.0)	0.4 (0.8)	1.0 (1.2)
Constipation	0.6 (1.0)	1.0 (1.2)	0.6 (0.9)	0.5 (0.9)	0.4 (0.8)	0.8 (1.2)
Sore of dry mouth	0.9 (1.1)	1.2 (1.2)	0.8 (1.0)	0.8 (1.0)	0.5 (0.9)	1.0 (1.1)
Drowsiness	1.1 (1.1)	1.5 (1.2)	1.0 (1.0)	0.9 (1.0)	0.6 (0.8)	1.2 (1.2)
Poor mobility	1.4 (1.3)	2.1 (1.2)	1.5 (1.2)	1.2 (1.2)	0.6 (1.0)	1.7 (1.4)
Itching	1.0 (1.2)	1.4 (1.3)	1.0 (1.1)	0.9 (1.1)	0.7 (1.0)	1.1 (1.2)
Difficulty sleeping	1.2 (1.3)	1.7 (1.3)	1.3 (1.2)	1.1 (1.2)	0.9 (1.2)	1.4 (1.4)
Restless legs	1.0 (1.2)	1.4 (1.3)	0.9 (1.1)	0.8 (1.1)	0.6 (1.0)	1.1 (1.3)
Changes in skin	0.7 (1.0)	1.0 (1.1)	0.7 (1.0)	0.7 (1.0)	0.5 (0.9)	0.9 (1.2)
Diarrhoea	0.5 (0.9)	0.6 (1.0)	0.5 (0.9)	0.4 (0.8)	0.3 (0.7)	0.7 (1.0)
Feeling anxious	1.0 (1.2)	1.6 (1.3)	1.0 (1.0)	0.8 (1.0)	0.5 (0.9)	1.2 (1.4)
Feeling depressed	0.9 (1.1)	1.5 (1.3)	0.9 (1.1)	0.7 (1.0)	0.4 (0.8)	1.2 (1.3)

*Note*: *EQ-5D-5L* EuroQOL Five Dimensions - 5 levels version, *POS-S Renal* Palliative care Outcome Scale-Symptom Renal, *SD* standard deviation

^a^The data presented are for participants who answered at least one item in the EQ-5D-5L and POS-S-Renal surveys. High scores indicate high symptom severity on the POS-S Renal and more problems with the items on the EQ-5D-5L

### Patient activation

Those receiving haemodialysis comprised a much larger proportion of individuals in PAM Level 1 (67.2%) compared to those in PAM Level 4 (28.4%). In contrast, transplant patients represented 11.9% of those in PAM Level 1 and 47.1% in PAM Level 4. For patients not on RRT, the proportion in each activation group was fairly similar.

In terms of age, the distribution in the highest and lowest activation groups differed, with those in the youngest age category (18–44 years) making up around a quarter of individuals in PAM Level 4 and only about half of that (12.3%) in PAM Level 1. Conversely, for the oldest age category (75+), a higher proportion was found in PAM Level 1 (28.6%) compared to PAM Level 4 (12.7%). For ethnicity, Asians appeared to be less activated than Whites, representing 15.7% of those in PAM Level 1 and only 4.9% in PAM level 4. In contrast, the proportion of white individuals was higher in PAM Level 4 (75.1%) than in PAM Level 1 (61.9%). Lastly, individuals in the most deprived quintile appear to be less activated, making up 36% of those in PAM Level 1 and only half that in PAM level 4 (18.6%).

Regarding HRQoL, mean scores for mobility in the EQ-5D-5L questionnaire ranged from 3.2 in PAM Level 1 to 1.7 in PAM Level 4, indicating that the latter self-reported fewer problems in this area (Table 2). This pattern was consistent across all dimensions. We observed the same trend for the 17 items assessing symptom burden i.e. the more activated groups had lower scores indicating fewer symptoms experienced by the individual. The mean scores for pain, for example, ranged from 1.6 for PAM Level 1 compared to 0.7 for PAM Level 4.

### Health-related quality of life

**Fig. 1:**
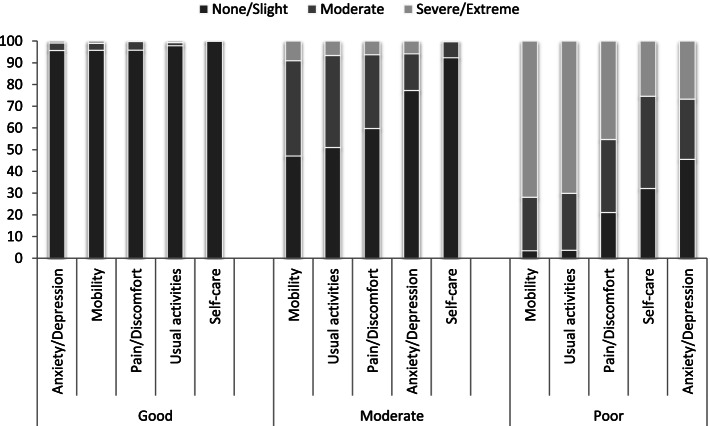
Latent class analysis for Health-Related Quality of Life

Results of the LCA established that a three-class solution provided a good fit for the HRQoL data as the BIC value reduced significantly from 2 to 3 classes, compared to 3 to 4 and 4 to 5 classes (BIC: 2 classes- 4017; 3 classes- 2987; 4 classes- 2747; 5 classes- 2723). Thus, when balancing fit and parsimony, we found that HRQoL is best separated into three distinct classes: ‘poor’, ‘moderate’, and ‘good’. The analyses showed that the proportion of the cohort in each latent class for HRQoL were as follows; ‘poor’ (27.4%), ‘moderate’ (35.5%) and ‘good’ (37.1%). For those in the ‘good’ class, over 95% of individuals reported experiencing no problems or slight problems across all dimensions of the EQ-5D-5L. Conversely, in the ‘poor’ class the majority of the patients reported moderate or severe problems (Fig. 1). This was more pronounced for mobility and usual activities with a larger proportion (> 70%) reporting severe or extreme problems compared to the other dimensions of HRQoL (< 50%).

### Symptom burden

**Fig. 2:**
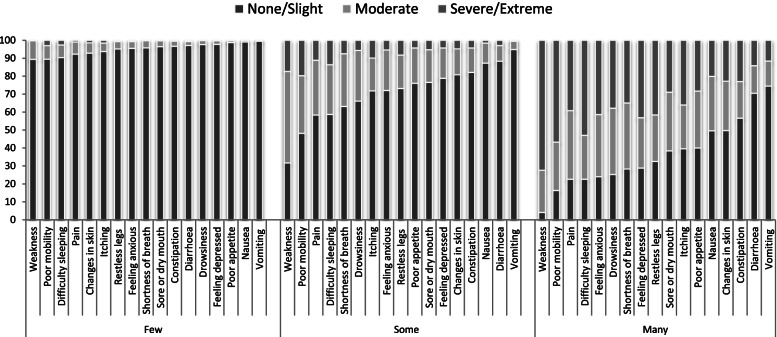
Latent class analysis for Symptom Burden

The LCA established a three-class solution for the symptom burden data. The proportion of patients classed as having ‘few’, ‘some’ and ‘many’ symptoms according to the LCA were 32.3, 46.2 and 21.5%, respectively. The analyses showed that over 89% of those in the ‘few’ symptoms class experienced no symptoms or slight symptoms across all dimensions. In contrast, the proportion of those in the ‘many’ symptoms class with severe or extreme symptoms appeared to vary widely across the different components of this measure, ranging from 72.4% for weakness and 11.6% for vomiting (Fig. 2).

### Association between patient activation and HRQoL and symptom burden

The multinomial regression analyses showed that patient activation was strongly associated with HRQoL. Participants in PAM Level 4 have much greater odds of reporting ‘good’ HRQoL (P = < 0.0001; OR 29.2, 95% CI 19.5–43.9) compared to those in PAM Level 1 (Table 3). A similar result was found for symptom burden with participants in PAM level 4 also having much greater odds of reporting few symptoms (P = < 0.0001; OR 25.9, 95% CI 16.8–40.2) compared to those in PAM Level 1. Stratification of the analyses by treatment type confirmed the association between higher patient activation and better HRQoL and reduced symptom burden (see Additional File 1 Table s3). Thus, as observed with the whole cohort, lower activation levels are associated with higher symptom burden and reduced HRQoL across treatment modalities. Due to small numbers in some of the categories in the stratified sample, the odds ratios for those in the peritoneal dialysis group are not presented in the table as these are unreliable.

**Table 3:** Examining factors associated with patient activation; specifically health-related quality of life and symptom burden

	Health-Related Quality of Life model		Symptom burden model	
	Good versus Poor	Moderate versus Poor	Few versus Many	Some versus Many
	Odds ratio (95% CI)	Odds ratio (95% CI)	Odds ratio (95% CI)	Odds ratio (95% CI)
**Patient activation (ref: PAM Level 1)**				
PAM Level 2	4.9 (3.5–6.8)	2.8 (2.2–3.6)	5.1 (3.6–7.3)	2.8 (2.2–3.8)
PAM Level 3	11.6 (8.5–15.7)	3.3 (2.6–4.2)	9.1 (6.6–12.5)	3.0 (2.4–3.9)
PAM Level 4	29.2 (19.5–43.9)	3.9 (2.7–5.8)	25.9 (16.8–40.2)	5.0 (3.4–7.5)
**Age** ^a^	0.98 (0.98–0.99)	1.00 (0.99–1.00)	1.02 (1.01–1.02)	1.02 (1.01–1.03)
**Gender (ref: Male)**				
Female	0.6 (0.5–0.7)	0.8 (0.7–1)	0.5 (0.4–0.7)	0.7 (0.6–0.9)
**Treatment type (ref: Haemodialysis)**				
Peritoneal dialysis	1.6 (0.9–2.8)	1.4 (0.9–2.4)	0.6 (0.4–1.2)	1.0 (0.6–1.6)
Non-dialysis	2 (1.5–2.6)	1.3 (1–1.6)	1.2 (0.9–1.7)	0.9 (0.7–1.2)
Transplant	2.2 (1.7–3)	1.3 (1–1.7)	2.7 (2–3.6)	1.3 (1–1.8)
**Deprivation** ^b^ **(ref: Quintile 1)**				
Quintile 2	0.7 (0.5–1.1)	1 (0.7–1.5)	0.9 (0.6–1.3)	0.8 (0.5–1.2)
Quintile 3	0.6 (0.4–0.9)	0.7 (0.5–1)	0.6 (0.4–0.9)	0.7 (0.4–1)
Quintile 4	0.4 (0.3–0.6)	0.7 (0.5–1)	0.6 (0.4–0.9)	0.8 (0.5–1.1)
Quintile 5	0.4 (0.3–0.6)	0.7 (0.5–0.9)	0.4 (0.3–0.6)	0.5 (0.3–0.7)
**Ethnicity (ref: White)**				
Asian	0.8 (0.5–1.1)	0.9 (0.7–1.3)	0.8 (0.5–1.1)	1.0 (0.8–1.5)
Black	1.1 (0.7–1.8)	1.1 (0.7–1.6)	1.4 (0.9–2.4)	1.2 (0.7–1.8)
Chinese	1.1 (0.2–5.5)	1.2 (0.3–5)	0.8 (0.2–3.6)	0.4 (0.1–2)
Other	1.2 (0.4–3.6)	1.5 (0.6–3.7)	0.8 (0.3–2.6)	1.2 (0.5–2.9)

^a^Values are odds ratios (95% confidence intervals), reflecting the odds of having a poor versus good and moderate health-related quality of life (HRQoL) and many versus few and some symptoms, per unit increase in age

^b^Deprivation quintile 1 is the least deprived group and quintile 5 is the most deprived group

## Discussion

To our knowledge, this is the first study to explore the association between PRO factors (i.e. HRQoL and symptom burden) and patient activation across the CKD spectrum. Our findings demonstrate a strong link between patient activation and HRQoL, and symptom burden, with individuals who are highly activated reporting a better HRQoL and a lower symptom burden. This study also highlights certain factors such as being older, on haemodialysis, deprived, or from a non-white background which are associated with reduced levels of patient activation.

Low activation (PAM Level 1 or 2) was reported by almost half of CKD patients, indicating that they are passive recipients in their healthcare and lack the knowledge and confidence to take action [28]. Other CKD studies have reported similar activation levels, with 38 to 46% classed as PAM Level 1 and 2 [29–33], whilst others have reported 60% [34]. Our findings suggest that people with CKD have lower activation when compared to those with other chronic conditions, including inflammatory bowel disease, diabetes, HIV, and multiple sclerosis [35–38].

In our sample, haemodialysis patients were the least activated group. This is consistent with similar studies that have reported low activation levels in over half of haemodialysis patients [39]. Our finding of lower activation levels in the haemodialysis population compared to the non-dialysis or transplant groups mirrors that of other studies [29, 31, 32, 40]. The high morbidity [39] and heavy symptom and treatment burden [41] associated with dialysis may impact the ability to undertake self-management tasks [42].

One of the key findings in our study was that the odds of having a better HRQoL and reduced symptom burden were much greater for patients who were more highly activated, after adjusting for age, gender, treatment modality, ethnicity, and deprivation. This is similar to previous studies which have shown an association between higher activation and a better QoL in older individuals with comorbidities [43], as well as patients with CKD [31, 34], multiple sclerosis [38] and inflammatory bowel disease [44].

To our knowledge, this is the first study to show an association between a reduced symptom burden and higher activation in CKD patients. An association between lower activation and worse self-reported health has previously been demonstrated in a CKD population [34] and a comorbid CKD and diabetic population [31]. This relationship may be driven by the fact that individuals who take an active role in their healthcare are more able to manage their symptoms and side effects. However, it is possible that individuals with a lower symptom burden feel more physically able to undertake self-management tasks [45]. Previous studies have demonstrated the impact of fatigue on haemodialysis patients’ abilities to complete daily activities [46]. Indeed, the importance of providing patients with support to complete daily tasks is further emphasised through our findings that mobility and the ability to perform usual activities appear to be the key determinants of HRQoL. However, due to the overlapping components of HRQoL and symptom burden we were not able to carry out mediation analysis to explore the direction of the relationship between these variables.

### Strengths and limitations

This study is strengthened by its large and nationwide sample of patients across different disease stages and treatment modalities, and as such it is the first study to present activation levels in a diverse kidney patient population including individuals on and off RRT. The utilisation of LCA allowed for assessment of heterogeneity in the HRQoL and symptom burden data and provided comprehensive evaluation of these measures in the study subjects based on all dimensions. Identifying subgroups in our data is important given that different groups of individuals in the population may require tailored support to increase patient activation levels.

As this was a secondary analysis of a national service evaluation programme, the recruitment strategy was not designed for research purposes and therefore no data is available for estimation of sample bias. Lack of Renal Registry information about patients who are not receiving RRT meant that it was not possible to know the CKD stage of the “non-dialysis” participants, and therefore no inference could be made about any association of CKD severity with the outcomes captured for this group. This study is also limited by our inability to adjust for comorbidity burden, despite it being well known that comorbidities have a great impact on HRQoL [47] as well on symptom burden [48]. This is primarily due to the reliability (or lack thereof) and completeness of the UKRR comorbidity data (mainly due to underreporting) to allow meaningful adjustment in this analysis. Another limitation is the cross-sectional study design which prevents the analysis of patient activation, HRQoL, and symptom burden over time.

In common with other studies involving self-reported data, we cannot exclude selection bias due to individuals with higher activation being more willing to participate and complete the outcome measures.

### Future work

This overlooked topic merits more research and clinical attention to optimise resource targeting and deliver improved care quality and outcomes at lower costs. As patient activation underpins effective and sustained self-management, its improvement provides an attractive goal for interventions aiming to promote and facilitate self-management behaviours. There is an urgent need to design, deliver and evaluate such interventions. Our observation of the association between higher activation levels and better patient-reported outcomes provides a potential engagement incentive for incorporation in such interventions, as patients often value these outcomes more than clinical benefits. Therefore, longitudinal studies should measure changes in activation over time to understand how patients transition from higher to lower activation, and how doing so impacts outcomes valued by the individual. Furthermore, future work should investigate whether symptom burden mediates the relationship between patient activation and QoL, including the possibility that improving patient reported outcomes could in itself lead to increased activation.

### Clinical relevance

This population, particularly individuals receiving haemodialysis, display lower activation levels than other chronic disease populations, and are in need of targeted self-management support. The association of PAM levels with PROs suggests that approaches aiming to improve activation may have the potential to impact aspects of health and life which are valued by the individual as well as by clinicians and healthcare provider organisations. Patient education, resources, support, and advice with symptom management and physical rehabilitation may be required to support self-management behaviours and facilitate the transition to higher activation states in this population. Systematic development and evaluation of educational and behavioural support strategies focussing on individual needs and priorities are warranted, and could usefully incorporate the potential for improving valued PROs to encourage patient engagement.

## Summary/conclusion

Patient activation was low in individuals with CKD across different treatment modalities, with nearly half of patients reporting low activation. Those who were highly activated had higher odds of better HRQoL and a lower symptom burden. Our findings warrant future studies into the impact of self-management support strategies on the patient experience as well as on clinical and cost outcomes.

## Supplementary Information

**Additional file 1 Table S1**. Baseline characteristics for participants who had missing data on all EQ-5D-5L dimensions or POS-S Renal items who were excluded from the analyses, presented with column percentages unless indicated otherwise. **Table S2**. Comparison of baseline characteristics of all prevalent CKD and RRT patients in the UK Renal Registry at 31st December 2016 and the English TP-CKD study cohort (values are numbers (% after excluding missing), unless indicated otherwise). **Table S3**. Multinomial regression analysis of the association between patient activation with health-related quality of life and symptom burden, stratified by treatment type.

## Data Availability

The data underlying this article were provided by the UK Renal Registry by permission. Data will be shared on reasonable request to the corresponding author with permission of the UK Renal Registry.

## Abbreviations

CI: Confidence interval
CKD: Chronic Kidney Disease
EQ-5D-5L: EuroQol 5 Dimension 5 Levels
HRQoL: Health-related Quality of Life
LCA: Latent Class Analysis
OR: Odds ratio
PAM: Patient Activation Measure
PRO: Patient Reported Outcome
POS-S Renal: Palliative care Outcome Scale – Symptoms Renal
RRT: Renal replacement therapy
TP-CKD: Transforming Participation in Chronic Kidney Disease
UKRR: United Kingdom Renal Registry
