# Supplementary material for: Patient activation and its association with symptom burden and quality of life across the spectrum of chronic kidney disease stages in England
Source: BMC Nephrol. 2022 Jan 26;23:45. doi: 10.1186/s12882-022-02679-w (PMC8793272; doi:10.1186/s12882-022-02679-w)
Supplement: Supplementary file 1 — Additional file 1 Table S1. Baseline characteristics for participants who had missing data on all EQ-5D-5L dimensions or POS-S Renal items who were excluded from the analyses, presented with column percentages unless indicated otherwise. Table S2. Comparison of baseline characteristics of all prevalent CKD and RRT patients in the UK Renal Registry at 31st December 2016 and the English TP-CKD study cohort (values are numbers (% after excluding missing), unless indicated otherwise). Table S3. Multinomial regression analysis of the association between patient activation with health-related quality of life and symptom burden, stratified by treatment type. [file 12882_2022_2679_MOESM1_ESM.docx]

**Table S1. Baseline characteristics for participants who had missing data on all EQ-5D-5L and/or POS-S Renal items who were excluded from the analyses, presented with column percentages unless indicated otherwise**

|  | **All** | **PAM Level 1** | **PAM Level 2** | **PAM Level 3** | **PAM Level 4** | **Missing** |
| --- | --- | --- | --- | --- | --- | --- |
| **Total N** | 303 (100) | 56 (100) | 30 (100) | 80 (100) | 39 (100) | 98 (100) |
|  |  |  |  |  |  |  |
| **Covariates** |  |  |  |  |  |  |
| **Treatment type** |  |  |  |  |  |  |
| Haemodialysis | 109 (36.0) | 28 (50.0) | 12 (40.0) | 20 (25.0) | 12 (30.8) | 37 (37.8) |
| Peritoneal dialysis | 9 (3.0) | 1 (1.8) | 2 (6.7) | 5 (6.3) |  | 1 (1.0) |
| Transplant | 102 (33.7) | 13 (23.2) | 7 (23.3) | 29 (36.3) | 16 (41.0) | 37 (37.8) |
| Non-dialysis | 83 (27.4) | 14 (25.0) | 9 (30.0) | 26 (32.5) | 11 (28.2) | 23 (23.5) |
|  |  |  |  |  |  |  |
| **Age (years)** |  |  |  |  |  |  |
| 18-44 | 28 (9.2) | 7 (12.5) | 1 (3.3) | 10 (12.5) | 6 (15.4) | 4 (4.1) |
| 45-54 | 54 (17.8) | 6 (10.7) | 6 (20.0) | 13 (16.3) | 8 (20.5) | 21 (21.4) |
| 55-64 | 60 (19.8) | 9 (16.1) | 5 (16.7) | 19 (23.8) | 9 (23.1) | 18 (18.4) |
| 65-74 | 83 (27.4) | 15 (26.8) | 9 (30.0) | 22 (27.5) | 8 (20.5) | 29 (29.6) |
| 75+ | 77 (25.4) | 19 (33.9) | 9 (30.0) | 16 (20.0) | 8 (20.5) | 25 (25.5) |
| Missing | 1 (0.3) |  |  |  |  | 1 (1.0) |
|  |  |  |  |  |  |  |
| **Gender** |  |  |  |  |  |  |
| Male | 142 (46.9) | 33 (58.9) | 10 (33.3) | 33 (41.3) | 14 (35.9) | 52 (53.1) |
| Female | 94 (31.0) | 12 (21.4) | 13 (43.3) | 26 (32.5) | 15 (38.5) | 28 (28.6) |
| Missing | 67 (22.1) | 11 (19.6) | 7 (23.3) | 21 (26.3) | 10 (25.6) | 18 (18.4) |
|  |  |  |  |  |  |  |
| **Ethnicity** |  |  |  |  |  |  |
| White | 193 (63.7) | 37 (66.1) | 19 (63.3) | 45 (56.3) | 24 (61.5) | 68 (69.4) |
| Asian | 18 (5.9) | 5 (8.9) | 2 (6.7) | 5 (6.3) | 3 (7.7) | 3 (3.1) |
| Black | 14 (4.6) | 3 (5.4) | 2 (6.7) | 3 (3.8) | 1 (2.6) | 5 (5.1) |
| Chinese | 3 (1.0) | 0 (0) | 0 (0) | 1 (1.3) | 0 (0) | 2 (2.0) |
| Other | 5 (1.7) | 0 (0) | 0 (0) | 3 (3.8) | 0 (0) | 2 (2.0) |
| Missing | 70 (23.1) | 11 (19.6) | 7 (23.3) | 23 (28.8) | 11 (28.2) | 18 (18.4) |
|  |  |  |  |  |  |  |
| **IMD** |  |  |  |  |  |  |
| Quintile 1 (least deprived) | 41 (13.5) | 9 (3.0) | 4 (1.3) | 11 (3.6) | 6 (2.0) | 11 (3.6) |
| Quintile 2 | 49 (16.2) | 8 (2.6) | 4 (1.3) | 14 (4.6) | 7 (2.3) | 16 (5.3) |
| Quintile 3 | 46 (15.2) | 8 (2.6) | 3 (1.0) | 7 (2.3) | 11 (3.6) | 17 (5.6) |
| Quintile 4 | 62 (20.5) | 13 (4.3) | 6 (2.0) | 22 (7.3) | 7 (2.3) | 14 (4.6) |
| Quintile 5 (most deprived) | 97 (32.0) | 17 (5.6) | 13 (4.3) | 24 (7.9) | 7 (2.3) | 36 (11.9) |
| Missing | 8 (2.6) | 1 (0.3) | 0 (0) | 2 (0.7) | 1 (0.3) | 4 (1.3) |
|  |  |  |  |  |  |  |
| **Outcomes** |  |  |  |  |  |  |
| EQ-5D-5L  Mean (SD) score | 2.1 (0.8) | 2.8 (0.8) | 2.2 (0.7) | 1.9 (0.7) | 1.6 (0.7) | 2.1 (0.7) |
| POS-S Renal  Mean (SD) score | 1.7 (0.5) | 1.9 (0.6) | 1.7 (0.5) | 1.6 (0.4) | 1.6 (0.6) | 1.6 (0.5) |
| Note. EQ-5D-5L, EuroQOL Five Dimensions (used to measure health-related quality of life)- 5 levels version; IMD, index of multiple deprivation; POS-S Renal: Palliative care Outcome Scale Symptom Renal (used to measure symptom burden); SD: standard deviation. | | | | | | |

**Table S2. Comparison of baseline characteristics of all prevalent CKD and RRT patients in the UK Renal Registry at 31st December 2016 and the English TP-CKD study cohort (values are numbers (% after excluding missing), unless indicated otherwise)**

|  | **Non-RRT** | | **Peritoneal dialysis** | | **Haemodialysis** | | **Transplant** | |
| --- | --- | --- | --- | --- | --- | --- | --- | --- |
|  | **Prevalent CKD and RRT cohort** | **TP-CKD**  **Study cohort¹** | **Prevalent CKD and RRT cohort** | **TP-CKD Study cohort** | **Prevalent CKD and RRT cohort** | **TP-CKD Study cohort** | **Prevalent CKD and RRT cohort** | **TP-CKD Study cohort** |
| **Total n** | **17,216** | **660** | **3,581** | **122** | **24,816** | **1,415** | **33,077** | **816** |
| **Gender (male)** | 9,406 (54.6) | 138 (61.9) | 2,147 (60) | 71 (62.3) | 15,262 (61.5) | 848 (61.1) | 20,038 (60.6) | 511 (62.8) |
| **Age (Mean, SD)** | 74.3, 13.6 | 63.1, 17.1 | 62, 15.9 | 62.6, 16.1 | 64.9, 15.3 | 64.4, 15.3 | 53.5, 13.8 | 54.3, 14.1 |
| **Ethnicity** |  |  |  |  |  |  |  |  |
| White | 12,076 (90.8) | 190 (91.8) | 2,578 (76.8) | 92 (81.4) | 16,623 (72.0) | 1,014 (73.9) | 24,917 (79.7) | 740 (91.5) |
| Asian | 837 (6.3) | 12 (5.8) | 414 (12.3) | 10 (8.8) | 3,115 (13.5) | 219 (16.0) | 3,510 (11.2) | 44 (5.4) |
| Black | 200 (1.5) | 4 (1.9) | 235 (7) | 9 (8.0) | 2,464 (10.7) | 119 (8.7) | 1,786 (5.7) | 16 (2.0) |
| Other | 184 (1.4) | 1 (0.5) | 130 (3.9) | 2 (1.8) | 888 (3.8) | 21 (1.5) | 1,051 (3.4) | 9 (1.1) |
| Missing | 3,919 | 452 | 224 | 8 | 1,726 | 35 | 1,813 | 2 |
| **Social deprivation** |  |  |  |  |  |  |  |  |
| IMD Quintile 1 (least deprived) | 3,867 (22.9) | 99 (15.7) | 571 (17.3) | 25 (21.0) | 2,915 (12.7) | 179 (12.7) | 5,518 (18.2) | 159 (19.5) |
| IMD Quintile 2 | 3,793 (22.4) | 131 (20.8) | 601 (18.2) | 28 (23.5) | 3,613 (15.7) | 187 (13.3) | 5,905 (19.5) | 160 (19.6) |
| IMD Quintile 3 | 3,680 (21.8) | 108 (17.1) | 671 (20.3) | 22 (18.5) | 4,418 (19.2) | 227 (16.1) | 6,024 (19.9) | 138 (16.9) |
| IMD Quintile 4 | 3,173 (18.8) | 124 (19.7) | 709 (21.5) | 18 (15.1) | 5,347 (23.3) | 293 (20.8) | 6,447 (21.3) | 190 (23.3) |
| IMD Quintile 5 (most deprived) | 2,397 (14.2) | 168 (26.7) | 747 (22.6) | 26 (21.8) | 6,685 (29.1) | 524 (37.2) | 6,371 (21.1) | 168 (20.6) |
| Missing | 306 | 30 | 282 | 3 | 1,838 | 5 | 2,812 |  |
| Note. Non-RRT: Non-renal replacement therapy (i.e. patients with chronic kidney disease not on renal replacement therapy), RRT: renal replacement therapy, SD: standard deviation, TP-CKD: Transforming Participation in chronic kidney disease, IMD: index of multiple deprivation area [26]  ¹The TP-CKD study cohort comprises CKD patients recruited from 14 renal units across England between December 2015 and December 2017, either on renal replacement therapy (RRT; dialysis or transplantation) or not on RRT (termed non-dialysis). | | | | | | | | |

**Table S3.** **Multinomial regression analysis of the association between patient activation with health-related quality of life and symptom burden, stratified by treatment type**

|  | **Total** | **Haemodialysis** | **Transplant** | **Non-dialysis** |
| --- | --- | --- | --- | --- |
|  | **Health-Related Quality of Life model** **(Good versus poor) Odds ratio (95% CI)** | | | |
| **Patient activation (ref: PAM Level 1)** |  |  |  |  |
| PAM Level 2 | 4.9 (3.5-6.8) | 5.4 (3.4-8.5) | 4.2 (1.9-9.6) | 5.6 (2.8-11.4) |
| PAM Level 3 | 11.6 (8.5-15.7) | 9.5 (6.3-14.3) | 15.3 (7.5-31.1) | 15.4 (7.8-30.2) |
| PAM Level 4 | 29.2 (19.5-43.9) | 18.8 (10.7-33.1) | 48 (20.6-111.9) | 43.2 (15.8-118.6) |
|  | **Symptom burden model** **(Few versus many) Odds ratio (95% CI)** | | | |
| **Patient activation (ref: PAM Level 1)** |  |  |  |  |
| PAM Level 2 | 5.1 (3.6-7.3) | 6.8 (4.2-11.1) | 3.1 (1.4-7) | 4.9 (2.3-10.5) |
| PAM Level 3 | 9.1 (6.6-12.5) | 7.3 (4.8-11.2) | 13.5 (6.4-28.3) | 10 (5-20) |
| PAM Level 4 | 25.9 (16.8-40.2) | 13.4 (7.2-24.9) | 52.4 (20.3-135) | 41.7 (14.6-118.9) |
| 1) The results for the peritoneal dialysis group have not been presented due to small numbers, yielding unreliable estimates. In addition, for the categories reported, some have large confidence intervals/ standard errors, therefore, please take caution when interpreting the strength of these relationships even though they results are significant.  2) The following variables were adjusting for in the model: age, gender, ethnicity and deprivation | | | | |
|  |  |  |  |  |
